# Melanomacrophage Centers As a Histological Indicator of Immune Function in Fish and Other Poikilotherms

**DOI:** 10.3389/fimmu.2017.00827

**Published:** 2017-07-17

**Authors:** Natalie C. Steinel, Daniel I. Bolnick

**Affiliations:** ^1^Department of Integrative Biology, The University of Texas at Austin, Austin, TX, United States; ^2^Department of Medical Education, Dell Medical School, The University of Texas at Austin, Austin, TX, United States

**Keywords:** melanomacrophage center, germinal center, fish immunology, non-model organisms, comparative immunology

## Abstract

Melanomacrophage centers (MMCs) are aggregates of highly pigmented phagocytes found primarily in the head kidney and spleen, and occasionally the liver of many vertebrates. Preliminary histological analyses suggested that MMCs are structurally similar to the mammalian germinal center (GC), leading to the hypothesis that the MMC plays a role in the humoral adaptive immune response. For this reason, MMCs are frequently described in the literature as “primitive GCs” or the “evolutionary precursors” to the mammalian GC. However, we argue that this designation may be premature, having been pieced together from mainly descriptive studies in numerous distinct species. This review provides a comprehensive overview of the MMC literature, including a phylogenetic analysis of MMC distribution across vertebrate species. Here, we discuss the current understanding of the MMCs function in immunity and lingering questions. We suggest additional experiments needed to confirm that MMCs serve a GC-like role in fish immunity. Finally, we address the utility of the MMC as a broadly applicable histological indicator of the fish (as well as amphibian and reptilian) immune response in both laboratory and wild populations of both model and non-model vertebrates. We highlight the factors (sex, pollution exposure, stress, stocking density, etc.) that should be considered when using MMCs to study immunity in non-model vertebrates in wild populations.

## Introduction

The study of immunology in wild vertebrates is hamstrung because many tools cannot readily be used in the field, and species-specific reagents do not exist for most non-model organisms. Melanomacrophage centers (MMCs) might offer a simple, cheap, and broadly applicable measure of adaptive immunity in poikilotherms. Here, we summarize and critically review this potentially valuable tool for wild immunology.

**Figure 1:**
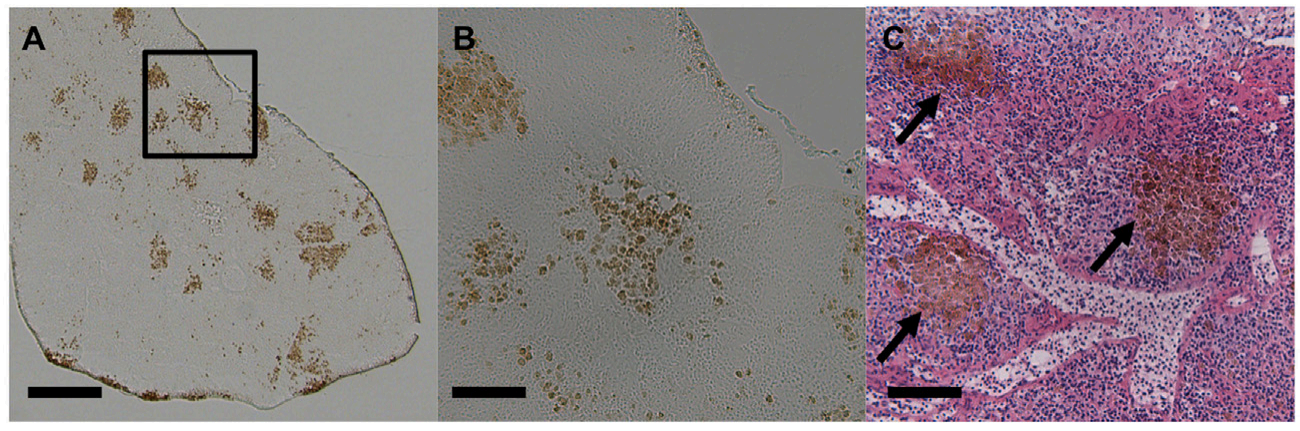
Light micrographs of stickleback (*Gasterosteus aculeatus*) splenic melanomacrophage centers. **(A)** Unstained spleen at 50×. Scale bar equals 250 µm. Box outlines magnified section in panel **(B)**. **(B)** Unstained spleen at 200×. Scale bar equals 62.5 µm. **(C)** H&E-stained spleen at 200×, and black arrows indicate MMCs. Scale bar equals 62.5 µm.

Melanomacrophages (or melanin-macrophages, MMs) are pigmented phagocytes found primarily in poikilotherm lymphoid tissues. MMs are darkly pigmented due to high lipofuscin, melanin, and haemosiderin content (1), making them histologically distinguishable *via* light microscopy (Figure 1). Nodular accumulations of closely packed MMs, known as MMCs, are primarily observed in the kidney, spleen, and liver. Generally, kidney and liver MMCs are diffuse and less structured, while splenic MMCs are more organized (2–4). As detailed below, the leading hypothesis is that MMCs represent a primitive site of adaptive immune system activation. As such, they may offer a valuable low-cost marker for measuring adaptive immunity across most of the vertebrate tree of life, in both model and non-model species. If so, MMCs could provide a widely applicable tool for wild immunology, as they are near-ubiquitous across vertebrates. MMCs are reported in over 130 fish species (Figure S1 and Table S1 in Supplementary Material) and are also present in amphibians and most reptiles (Figures S2 and S3 and Table S2 in Supplementary Material). This review centers on the well-studied piscine MMC but comments on the less-well known amphibian and reptile MMCs, where relevant.

## Non-Immunological Functions of the MMC

The MMC is thought to play dual roles, participating both in immune defenses and normal, non-immunological, physiological processes. This review focuses on MMC immune functions but touches briefly on non-immunological roles [detailed review by Wolke (5)]. Like other macrophages, MMs’ primary function is phagocytosis. The presence and long-term storage of unmetabolized, effete materials, earned MMCs the title of “metabolic dumps” (6). This indigestible material, which gives MMs their characteristic pigmentation, can be of endogenous or exogenous origins. Endogenous materials are obtained through the phagocytosis of exhausted cells, particularly erythrocytes. Erythrophagocytosis by MMCs is widely reported and goes back to the earliest MMC description by Blumenthal in 1908 (7–13). More recent studies showed that turtle MMs can erythrophagocytose *in vitro* (14). The presence of degraded erythrocytes and hemosiderin suggests that MMs function in iron recycling (10, 11), much like the hemosiderin-laden splenic red pulp macrophages (RPMs) found in mammals (15). Exogenous materials, of natural or experimental origins, also collect within MMCs. Metal deposits (16, 17) and experimentally injected inert substances (13, 18–23) accumulate within MMs. These findings highlight the importance of MMCs in debris clearance and long-term storage of highly indigestible and/or toxic materials. The phagocytic nature of these cells is similar to that of the tingible body macrophage (TBM) found in the mammalian splenic germinal center (GC) (24). While the role of waste product repository is considered non-immunological, as we discuss in the following section, this physiological necessity may overlap with an important MMC immunological function: antigen retention.

## Immune Functions of the MMC

Early descriptions proposed that MMCs function in both the innate and adaptive arms of the immune response (25). MM phagocytic activity is not limited to erythrocytes as they also phagocytose infectious materials (14, 21, 25–27). Turtle MMs are described as “aggressively phagocytic,” attacking bacteria, fungi, and helminth parasite eggs *in vitro* (14). MMCs’ close association with specialized capillaries in the spleen, known as ellipsoids (8, 28), suggests that they may scavenge blood borne pathogens. This notion is supported by the observation that, *in vivo*, MMCs quickly remove injected foreign materials from the circulation (13, 18–20, 27).

Morphological characteristics, organ location, and association with infection/immunization led to the hypothesis that MMCs are analogs, or “primitive” evolutionary precursors, of the mammalian GC (1, 5, 10, 21, 27–32). As such, they may participate in the adaptive immune response. In mammals, the GC response is crucial for the differentiation and clonal expansion of memory B cells and high-affinity plasma cells. Extensive experimentation has elucidated the complex spatial structure, cell–cell interactions, and molecular processes that occur within the mammalian GC (33). The GC has a well-defined architecture, with distinctive B-cell, follicular dendritic cell (FDC) and T follicular helper cell (T_FH_) aggregates. Following antigen challenge, antigen-specific B cells accumulate and proliferate causing a transient increase in GC size (33). During the GC response, antigen-specific B cells, mediated by interactions with FDCs and T_FH_, undergo clonal expansion and differentiate into memory and plasma cells (33). FDCs serve as long-term antigen depots by maintaining intact antigen on their surface in the form of immune complexes (IC) (34). It is within GCs that antibody affinity maturation occurs. In this targeted microevolutionary process, immunoglobulin (Ig) genes undergo somatic hypermutation (SHM) and selection. In GC B cells, the enzyme activation-induced cytidine deaminase (AID) generates Ig gene mutations (35–37). GC B cells expressing mutated Ig genes are then selected based on their affinity for antigen (38, 39). This process hones antibody specificity and is necessary for an efficient humoral immune response.

Teleosts lack GCs; yet, nevertheless, they generate affinity-matured antibody in response to antigen challenge (40, 41). Descriptive studies, across many fish species, identified numerous similarities between GCs and MMCs (Table 1), raising the possibility that MMCs are the site of the teleost humoral adaptive immune response. MMs, like mammalian FDCs, stain positively with the CNA-42 monoclonal antibody (42) and express CSF1-R (43). Fish injected with both infectious and non-infectious substances demonstrated that MMCs are sites of antigen retention (18, 19, 21, 22, 30, 44–46). Notably, carp immunized with *Aeromonas hydrophila* retained antigen in and around splenic MMCs for at least a year (21). Antigen retained near or within MMCs is extracellular, trapped within IC, and the injection of preformed IC accelerates this retention (47, 48). While these findings highlight many similarities between MMs and FDCs, the erythrophagocytic and scavenging functions described in the previous section also suggest similarities between the MMC and RPMs and TBMs found in the mammalian spleen (Table 1).

**Table 1:** Comparison of poikilotherm melanomacrophages with mammalian follicular dendritic cells (FDC), red pulp macrophages (RPM), and tingible body macrophages (TBM).

	Mammals			Poikilotherms
	FDC	RPM	TBM	MMC
Location	Spleen, LN	Spleen	Spleen, LN	Spleen, kidney, liver
Nodular aggregations	+	−	−	+
Erythrophagocytosis	−	+	−	+
Phagocytosis of exhausted/dead cells	−	−	+	+
Stain with CNA-42	+	−	−	+
Express CSF1-R	+	−	−	+
Retain antigen long-term within ICs	+	−	−	+
Found in close proximity to:				
Lymphoid cells	+	−	+	+
AID-expressing cells	+	−	+	+
B cells undergoing SHM	+	−	+	Unknown
Differentiating B cells	+	−	+	Unknown
Activated B cells	+	−	+	Unknown

*LN, lymph node; IC, immune complexes; AID, activation-induced cytidine deaminase; SHM, somatic hypermutation*.

Following immunization or infection, fish MMCs increase in size and/or number (10, 13, 26, 32, 49–52), much like mammalian GCs. Study of MMC kinetics in goldfish showed that MMs can both join existing aggregates or form new aggregates (22). Lymphoid cells are observed in close proximity to teleost MMCs (13, 21, 53) and increase in response to immunization (13). These studies, however, lacked species-specific reagents; so determination of cell identity was not possible. More recent immunohistochemical studies revealed MMC-adjacent Ig+ cells that increase in number in response to experimental infection (2–4). These Ig+ cells were presumed to be B cells, but the cells’ identity has not been confirmed as these stains cannot differentiate between cells expressing Ig and those binding soluble Ig (or IC) on their cell surface (2–4). While some Ig+ cell aggregation was observed, they did not exhibit the highly compartmentalized structure characteristic of mammalian GCs (2–4). Unlike the GC, the MMC response in some teleost species does not correlate with an increased antibody production (13). More recently, AID-expressing cells were identified in or proximal to MMCs (54). Considering that AID expression is required for SHM in mammals, this finding strongly supports the notion of a GC-like MMC. However, SHM has not been directly documented in MMCs (54). These studies provide compelling evidence that MMCs and mammalian GCs likely perform similar functions, but differences do remain. A systematic investigation of MMC function has never been performed. Toward the end of this review, we suggest an experimental road map to fully characterize teleost MMC function.

## Evolutionary History of MMCs

**Figure 2:**
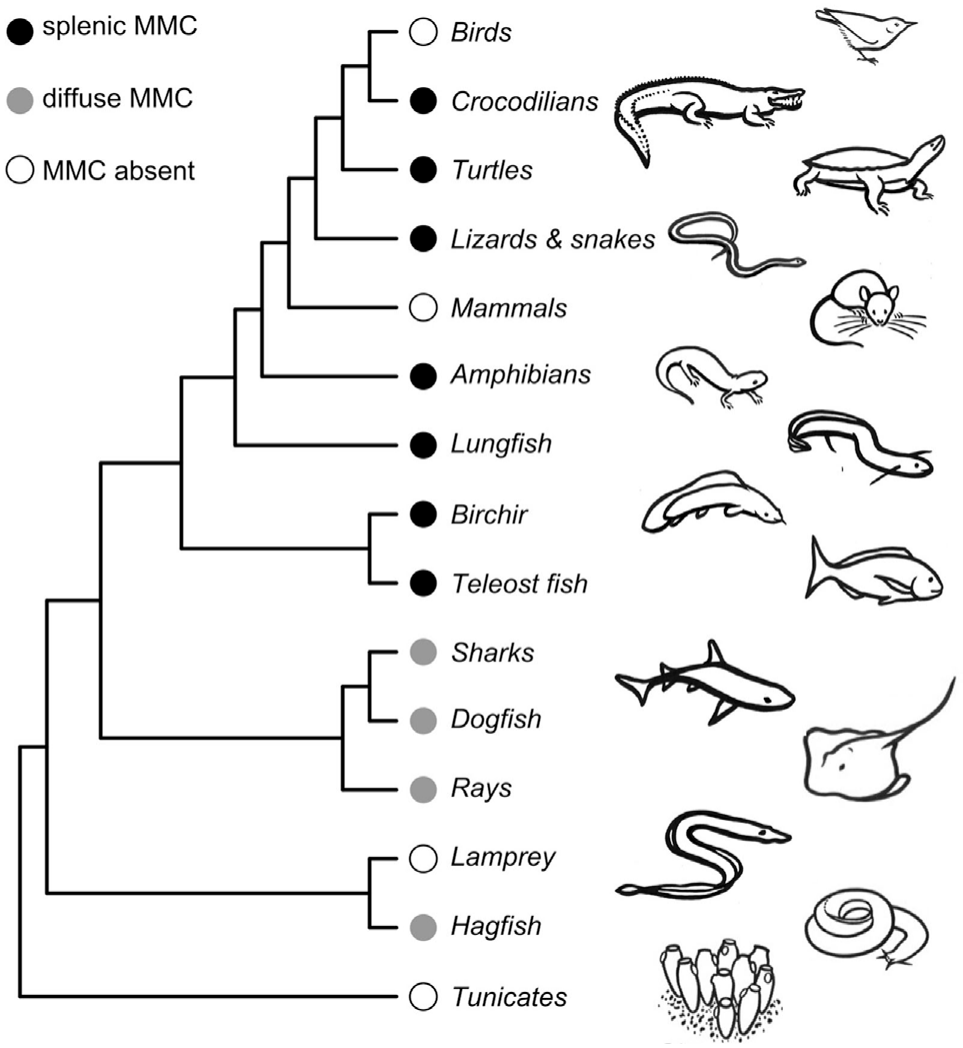
Phylogeny showing melanomacrophage center (MMC) gains and losses across vertebrate species. Detailed phylogenies of fish, amphibian, and reptilian species can be found in Supplemental Material. The phylogeny is not time-calibrated and was plotted using the *ape* package in R (57), using a topology obtained from the OneZoom database (58). Drawings by Doreen J. Bolnick.

Here, we consider both the likely timing and function of the early evolution of MMCs. MMCs are present in both jawless (Cyclostomata) and jawed vertebrates (Gnathostomata), implying that MMCs likely evolved at least 525 mya in a common ancestor of all Vertebrata (Figure 2). This evolutionary gain likely proceeds the evolution of adaptive immunity as jawed and jawless vertebrates evolved unique adaptive immune systems (55, 56). In “primitive” vertebrates like hagfish (Myxiniformes) and sharks and rays (Chondrichthyes), MMs are diffusely distributed, mostly in liver tissue. In contrast, bony vertebrates [Euteleostomi (a.k.a., Osteichthyes)] exhibit more aggregated MMs that form distinct MMCs primarily in the spleen and kidney (6). Because bony fish, amphibians, and reptiles share splenic aggregations, we infer that MMC concentration into the spleen must have evolved in a common ancestor to Euteleostomi. That is, splenic MMCs evolved between 430 and 460 mya, prior to the split between ray-finned fishes (Actinopterygii) from lobe-finned fishes and tetrapods (Sarcopterygii) but after their split from Chondrichthyes (Figure 2). Note that this inference is based on an assumption that MMCs are homologous structures (inherited from a common ancestor) rather than having independently evolved multiple times. This assumption has yet to be rigorously tested with transcriptomic and genetic data.

Melanomacrophage centers are retained in most poikilotherms, though they are reported absent in a handful of species. Admittedly, studies that did not locate MMCs (despite appreciable investigation) might be false negatives. Studies of MMCs in jawless vertebrates are underrepresented, with reports from only two Cyclostomata species (6). MMCs are reportedly absent in the lamprey, *Lampetra fluviatilis*, but present in hagfish (6) (Figure 2; Tables S1 and S3 in Supplementary Material). We are aware of only two bony fish species that reportedly lack MMCs: the fifteenspine stickleback, *Spinachia spinachia*, and the South American armored catfish, *Hypostomus francisci* (Figure S1 and Table S3 in Supplementary Material) (8, 59). In all three instances, MMC absence was determined by survey studies of a few individuals from a single location. From these limited studies, it is difficult to determine if the absence of MMCs represents species- or population-level variation. These absences should be investigated in greater detail but suggest that MMCs can be lost (or, replaced by another feature) without lethal effect. The sister genus to *Spinachia* (*Gasterosteus*, including threespine stickleback) has distinct MMCs (Figure 1), indicating that loss can occur over comparatively short macro-evolutionary time. MMCs have also been lost, probably two separate times, in Squamate reptiles (in vipers and a large snake clade including rat snakes and keelbacks) (Table S3 in Supplementary Material). But, MMCs were located in all amphibians we found reports for (Figure S2 in Supplementary Material), and in turtles, lizards, and crocodiles (Figure S3 in Supplementary Material). These aggregates are generally less organized than those observed in bony fish. MMCs are not observed in mammals or birds. If MMCs are indeed absent in birds and mammals, it is likely that they were independently lost. MMC loss in mammals is particularly note-worthy, because pigmented RPMs are still found in mammalian spleens. These macrophages perform physiological roles in erythrophagocytosis and metabolic recycling but have no known role in the GC response.

Collectively, evidence suggests several intriguing hypotheses for the evolutionary origins of MMs, MMCs, and mammalian GCs. We emphasize that these hypotheses are tentative but intriguing enough to warrant rigorous evaluation. The first hypothesis is that MMs initially evolved as molecular and cellular “garbage dumps” without a particular immunological role, in a common ancestor of all Vertebrata. Early MMs would have encountered pathogens or their antigens while acting in this clean-up capacity, which leads to a second hypothesis: MMs’ incidental interaction with pathogens subsequently became entrenched and they gained immune function, perhaps in a common ancestor to Gnathostomata. The third (and oldest) hypothesis is that in early mammals, the MMCs evolved into GCs, with splenic RPMs remaining to carry out erythrocyte recycling function. We consider this third hypothesis in greater detail in the next section.

## Are MMCs an Evolutionary Precursor to the Mammalian GC?

The notion of a “primitive GC”-like MMC has long been speculated (1, 5, 10, 21, 25, 27–32). However, this conclusion was drawn from descriptive studies, across many fish species, which did not directly investigate how MMC parameters correlate with immune function. As noted earlier, similarities between MMCs and GCs make this conclusion compelling (Table 1). Chiefly, both structures increase in size and/or number in response to immunization or infection (10, 13, 26, 31, 50–52). The structures also share cell types and expressed genes, but other observations call the “GC-precursor” hypothesis into question. Most notably, MMCs also respond to non-infectious settings, including poor body condition, pollution exposure, starvation, aging, and injury (discussed in detail below) (1, 10, 50, 60–63). When reconciling the MMC response to both infectious and non-infectious circumstances, these findings could be interpreted in several different ways.

First, it is possible that MMCs evolved from a purely physiological role, to gain immunological function, conducting both roles simultaneously. Subsequent GC evolution may be a case of sub-functionalization, in which a generalist tissue or cell type, performing multiple roles, evolves into specialist subpopulations. Second, the conditions that influence MMC status, including infection/immunization, are all associated with tissue destruction. So it is possible that the MMC response may not be immunological like the GC but may simply be a non-specific expansion of MMs in response to generalized tissue damage. In this case, the proposed immune function of MMCs may be a false lead.

Third, the association between MMCs and stress or tissue damage could be confounded with infection. Poor body condition, aging, injury, pollution exposure, and starvation can facilitate secondary infections. Thus, experiments that manipulate stressors might have incidental effects on infection, making it difficult to experimentally distinguish between non-infectious versus infectious MMC responses. Conversely, infection or pollution exposure may lead to poor body condition, injury, and/or suppressed appetite/starvation. As exposure to pathogens or contaminants may not be readily detectible, an MMC response may mistakenly be attributed to other environmental or physiological factors.

## Future Directions

To determine whether MMCs function in a GC-like capacity, further studies must directly test this hypothesis. Antigen-specific B-cell clonal expansion and high-affinity antibody production from mammalian GCs are the result of B-cell proliferation, differentiation, and SHM. To determine if MMCs are GC analogs, experiments must investigate if antigen-specific B cells proliferate and differentiate in association with MM aggregates. While the presence of AID-expressing cells within the MMC implies that affinity maturation is occurring (54), further experiments are necessary to assess whether antibody gene SHM occurs within and is dependent on the MMC. The mammalian GC response is T-cell dependent, as GC B-cell clonal expansion and differentiation requires help from T_FH_ cells (64). T-dependent (TD) antigens are proteins that induce GC reactions. T-independent (TI) antigens, in contrast, are polysaccharide based, cannot be presented to T_FH_ cells, and consequently do not stimulate a GC response (65). Therefore, a straightforward test of MMC function would compare the MMC response to TD and TI antigens. Using next generation sequencing tools, it would be useful to conduct a comparative transcriptomic analysis of MMCs and GCs to determine similarities and differences in gene expression. Lastly, MMC fine-scale structure should also be defined, for instance using single molecule fluorescent *in situ* hybridization to generate spatially explicit maps of gene expression and the distribution of cell types within MMCs. In the absence of functional studies that directly test the hypothesis of a GC-like MMC, an abundance of caution should be used when drawing conclusions regarding the nature of the MMC response in immune function.

## Notes for the Wild Immunologist

Though fundamental experiments are needed to clarify the immunological significance of the MMC, we nevertheless assert that the MMC is potentially a practical biomarker of immune function in both laboratory and wild studies. As MMCs are evolutionarily conserved in many poikilothermic species, histological assays of the MMC state could provide a valuable tool for comparative studies of adaptive immunity across the diversity of vertebrates. Due to their inherent pigmentation, the MMC response can be easily visualized and quantified *via* light microscopy, without the need for costly species-specific reagents. This pigmentation also makes MMs highly autofluorescent, a feature that can be employed to isolate MMs *via* FACS sorting (43, 54). Though MMCs appear to be a useful bioindicator of poikilotherm immune function, several variables must be considered when designing and analyzing laboratory or wild studies.

Careful consideration should be given to sampling relevant tissues and quantifying the appropriate MMC parameters. Most immunological (and non-immunological) studies of the MMC focus on a single tissue. In light of the phylogenetic association of the MMC with different organs (6), researchers should be sure that the appropriate tissue(s) are sampled. The MMC response is quantified using various parameters including aggregate size, number, total pigmented area, pigmentation intensity, and aggregate circularity (shape factor). These variables can change through time in response to various stressors, and some are correlated (e.g., size and pigmented area). However, in the absence of a functional understanding of the nature of the MMC in the immune response it’s not yet clear which metric(s) should be reported. Therefore, to avoid cherry-picking results, attention and justification should be given for choosing tissues to sample and MMC parameters to report.

The MMC can also respond to physiological and environmental changes. This presents a difficult situation for the wild immunologist. Histological MMC parameters vary in response to life history and environmental factors. Sex (60, 66), diet (52, 61, 63, 67), spawning phase (68), season (69), temperature (70), and UV exposure (71) influence MMC status. Several authors reported a linear correlation between age and elevated MMC metrics (17, 72–74). Studies also showed MMC responses to environmental stressors. In fish, farming increased MMC density compared to the same species raised in wild conditions (75). Other aquaculture variables, such as ranching time and stocking density, influence MMC metrics (67, 76).

Numerous investigations report correlations between “degraded environments” and MMC status. Decreased dissolved oxygen levels were associated with an increased fish MMC number (77). Several studies documented MMC responses to environmental contaminant exposure in wild fish and amphibians (66, 73, 77–81). However, many of these studies compared contaminant-exposed animals to those from uncontaminated “control” areas, without accounting for confounding effects of infection or other physiological or environmental factors. Nevertheless, controlled experimental exposure of lab-raised fish to environmental contaminants supports the notion of a pollutant-induced MMC response (52, 82–85). These observations, combined with MMC responses to life history and environmental factors (1, 10, 17, 31, 49, 50, 60–63, 72–74, 80), underscore the difficulty in interpreting MMC metrics in wild populations. If meaningful conclusions are to be drawn regarding the MMC response in wild (and lab-raised) animals, thoughtful consideration must be given to choosing appropriate controls and accounting for physiological and environmental variables in analyses. Therefore, we recommend wild studies employing the MMC assay without validation and appropriate controls be interpreted with caution.

## Conclusion

The MMC shares many structural, cellular, and molecular similarities with the mammalian GC, suggesting an evolutionary tie to mammalian adaptive immunity. Parallels between these structures led researchers to recognize the potential for the MMC as a histological biomarker of poikilotherm immune response. We assert, however, that this tool should be used with caution. While descriptive studies have identified important features of the MMC, functional studies are needed to confirm its role in the adaptive immune system. If such studies validate this immunological tool, they will pave the way for comparative studies of the evolutionary origins of vertebrate immunity and for experimental immunology in wild populations.

## Author Contributions

NS and DB wrote and edited the manuscript.

## Conflict of Interest Statement

The authors declare that the research was conducted in the absence of any commercial or financial relationships that could be construed as a potential conflict of interest.

## Abbreviations

MM, melanomacrophage; MMC, melanomacrophage center; GC, germinal center; RPMs, red pulp macrophages; TBMs, tingible body macrophages; FDC, follicular dendritic cell; T_FH_, T follicular helper cell; Ig, immunoglobulin; SHM, somatic hypermutation; AID, activation-induced cytidine deaminase.
