# Supplementary material for: Melanomacrophage Centers As a Histological Indicator of Immune Function in Fish and Other Poikilotherms
Source: Front Immunol. 2017 Jul 17;8:827. doi: 10.3389/fimmu.2017.00827 (PMC5512340; doi:10.3389/fimmu.2017.00827)
Supplement: Supplementary file 1 [file Table_1.pdf]

Supplementary Material

Melanomacrophage Centers as a Histological Indicator of Immune Function in Fish and Other Poikilotherms.

Natalie C. Steinel *, Daniel I. Bolnick

*** Correspondence:** Natalie C. Steinel: nsteinel@austin.utexas.edu

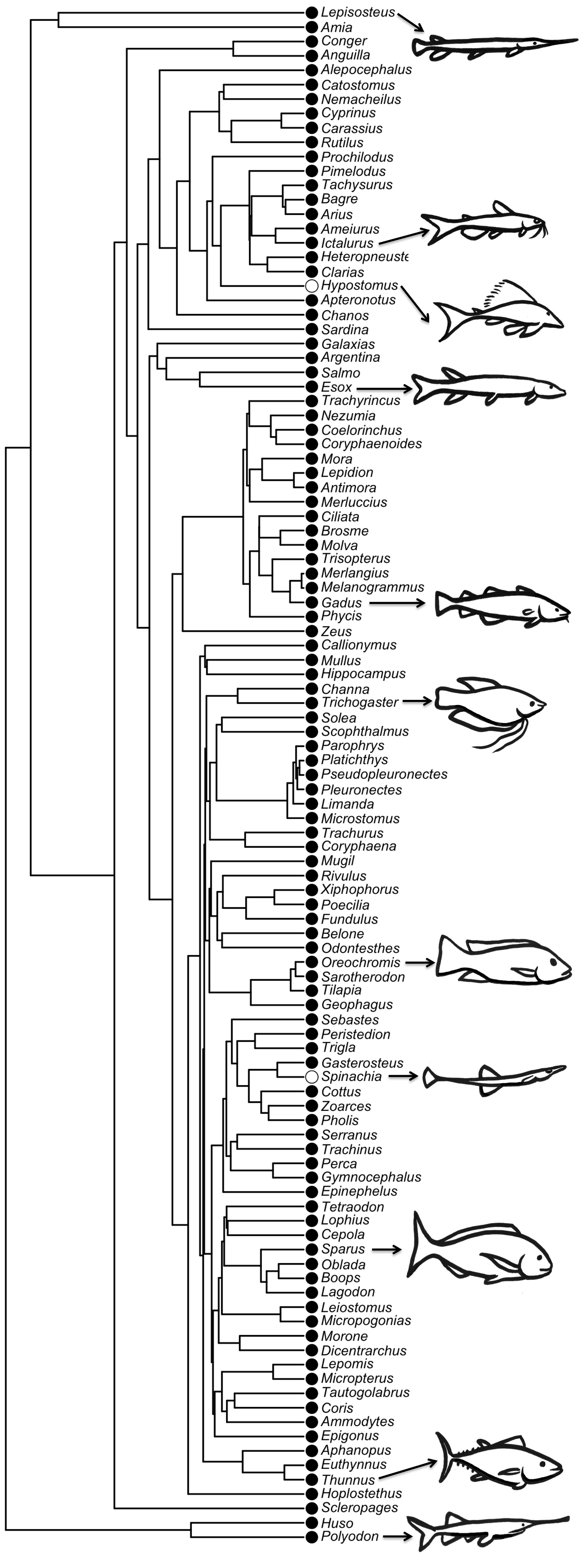

Supplemental Figure 1. Phylogeny of fish species for which MMCs status has been reported. Black circle indicates the presence of splenic, renal, and/or hepatic MMCs. White circle indicates MMCs have been reported absent. Drawings by Doreen J. Bolnick. The phylogeny is a subset of a time-calibrated large molecular phylogeny of ray-finned fishes (1), showing only those genera for which we have data on MMC presence or absence. The phylogeny was plotted using the *ape* package in R (2).

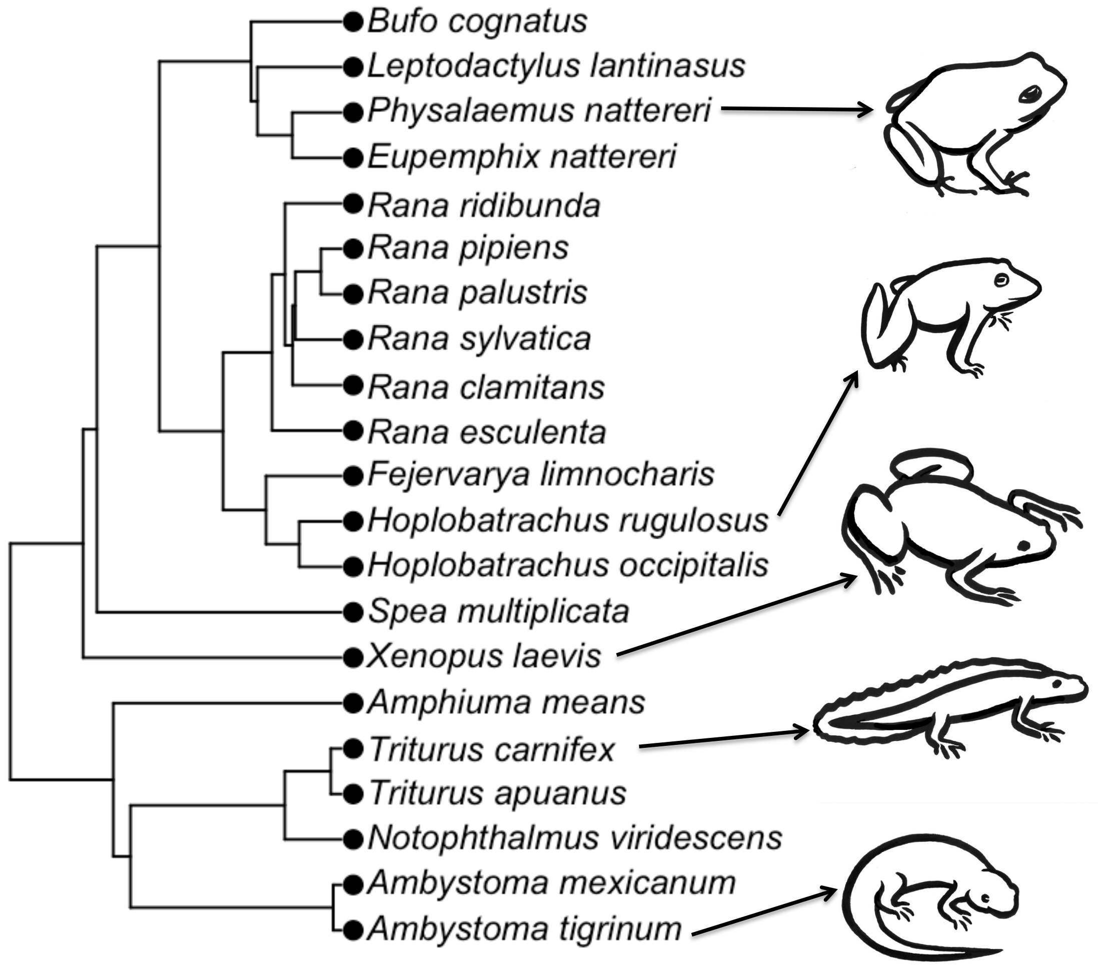

Supplemental Figure 2. Phylogeny of amphibian species for which MMC status has been reported. Black circle indicates the presence of splenic, renal, and/or hepatic MMCs. White circle indicates MMCs have been reported absent. Drawings by Doreen J. Bolnick. The phylogeny is a subset of a large time-calibrated molecular phylogeny of amphibians (3), showing only those species for which we have data on MMC presence or absence. The phylogeny was plotted using the *ape* package in R (2).

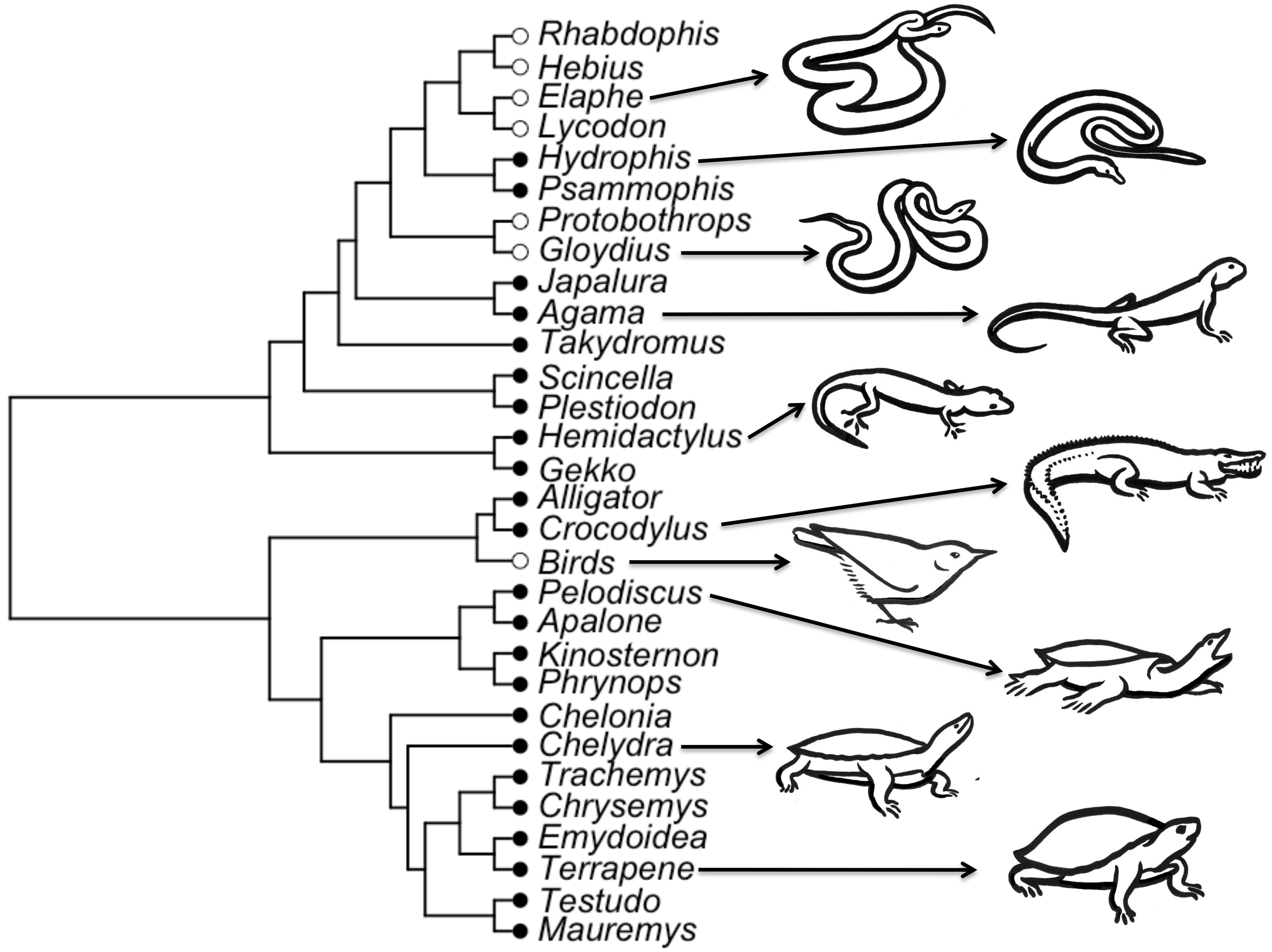

Supplemental Figure 3. Phylogeny of reptilian species for which MMC status has been reported. Black circle indicates the presence of splenic, renal, and/or hepatic MMCs. White circle indicates MMCs have been reported absent. Drawings by Doreen J. Bolnick. The lizard and snake phylogeny is a subset of a large time-calibrated molecular phylogeny of squamate reptiles (4), showing only those genera for which we have data on MMC presence or absence. We combined this tree with a non-time-calibrated tree topology of non-squamate reptiles obtained from the OneZoom database (5). The phylogeny was plotted using the *ape* package in R (2).

Supplemental Table 1. Fish species with MMCs reported in the spleen, kidney, and/or liver. +: MMCs present in tissue, -: MMCs absent in tissue, *: MMC status has not been determined for this tissue. §: MMCs have also been reported in the thymus of *Epinephelus malabaricus*.

Species	Common name	Spleen	Kidney	Liver	References
Alepocephalus abairdii	Baird's slickhead	+	+	+	(6)
Ameiurus nebulosus	brown bullheaded catfish	+	*	*	(7)
Amia calva	bowfin	+	+	+	(6)
Ammodytes	sand lance genus	+	*	*	(8)
Anguilla anguilla	European eel	+	+	+	(6,9,10)
Antimora rostrata	blue antimora	+	+	+	(6)
Aphanopus carbo Lowe	black scabbard fish	+	+	+	(6,9)
Apteronotus (formerly *Apteryna)*	knifefish genus	+	*	*	(8)
Argentina silus	Atlantic argentine (smelt)	+	+	+	(6)
Arius felis	hardhead catfish	+	*	*	(7)
Bagre marinus	gafftopsail catfish	+	*	*	(7)
Barbus conchonius	rosy barb	+	+	*	(11)
Belone belone gracilis	garfish	+	+	+	(6)
Boops boops	bogue	+	+	+	(6,9)
Brosme brosme	cusk	+	+	+	(6,9)
Callionymus lyra	dragonet	+	*	*	(12)
Callionymus maculatus	spotted dragonet	+	+	-	(6)
Carassius auratus	goldfish	+	+	+	(6,13-16)
Catostomus commersoni	white suckers	+	+	+	(17)
Cepola rubescens	red bandfish	+	+	+	(6)
Chalinura meiterranea	Mediterranean grenadier	+	+	+	(6)
Channa punctata	Indian spotted snakehead	+	+	*	(18)
Channa striata	Snakehead murrel	+	+	+	(6)
Chanos chanos	milkfish	+	+	+	(6)
Chimaera monstrosa	rabbit fish	-	-	+	(6)
*Ciliata (*formerly *Motella)*	lotids genus	+	*	*	(8)
Clarias gariepinus	African catfish	+	+	+	(19)
Coelorhynchus occa	spear-snoted grenadier	+	+	+	(6)
Conger	conger genus	+	*	*	(8)
Conger conger	European conger	+	+	+	(6)
Coris julis	Mediterranean rainbow wrasse	+	+	+	(6)
Coryphaena hippurus	mahi mahi	+	+	-	(6)
Coryphaenoides rupestris Gunnerus	roundnose grenardier	+	+	+	(6,9)
Cottus	sculpin genus	+	*	*	(8)
Cyprinus carpio	carp	+	+	*	(11,20-23)
Dicentrarchus labrax	sea bass	+	+	+	(24,25)
Epigonus telescopus	black cardinal fish	+	+	+	(6)
Epinephelus malabaricus §	malabar grouper	+	+	*	(26)
Esox lucius	pike	+	+	-	(6)
Euthynnus alletteratus	little tunny	+	*	*	(27)

Fundulus heteroclitus	mummichog	+	*	*	(28)
Gadus luscus	whiting pout	+	*	*	(12,13)
Gadus merlangus	whiting	+	*	*	(12)
Gadus minutus	poor cod	+	*	*	(12)
Gadus morhua	Atlantic cod	+	+	-	(6)
Gadus morrhuae	common cod	+	*	*	(12)
Gadus pollachius	pollack	+	*	*	(12)
Galaxias auratus	golden galaxias	+	+	-	(6)
Gasterosteus aculeatus	threespined stickleback	+	+	+	(6)
Geophagus brasiliensis	pearl cichlid	+	*	+	(29)
Gymnocephalus cernua	ruffe	+	*	+	(30)
Heteropneustes fossilis	catfish	*	+	*	(31)
Hippocampus kuda	sea horse	*	+	*	(32)
Hoplias malabaricus	wolf fish/tiger fish/trahira	+	+	+	(29,33)
Hoplostethus	slimehead genus	+	+	+	(6)
Huso huso	beluga (sturgeon)	-	-	+	(6)
Ictalurus furcatus	blue catfish	+	*	*	(7)
Ictalurus punctatus	channel catfish	+	+	+	(6,34)
Lagodon rhomboides	pinfish	+	*	*	(7)
Leiostomus xanthurus	spot	+	*	*	(7)
Lepidion eques	North Atlantic codling	+	+	+	(6)
Lepisosteus platostomus	shortnose gar	+	-	+	(6)
Lepomis macrochirus	bluegill	+	+	+	(6)
Limanda limanda	common dab	+	*	*	(12,35,36)
Lophius piscatorius	angler fish	+	+	-	(6,12)
Melanogrammus aeglefinus	haddock	+	+	+	(6)
Merlangius merlangus	whiting	+	+	+	(6,37)
Merluccius merluccius	hake (herring hake)	+	+	+	(6)
Micropogonias undulatus	Atlantic croaker	+	*	*	(7,28)
Micropterus salmoides	largemouth bass	+	+	+	(6,38,39)
Microstomus pacificus	Dover sole	*	*	+	(40)
Molva molva	ling	+	*	*	(12)
Mora moro	common mora	+	+	+	(6,9)
Morone americana	white perch	*	*	+	(41)
Morone labrax	salmon bass	+	*	*	(12)
Morone saxatilis	striped bass	+	*	+	(28,41)
Mugil	mullet genus	+	+	+	(6,9)
Mullus barbatus	red mullet	+	+	+	(6)
Myxine glutinosa	hagfish	-	-	+	(6)
Nezumia aequalis	common Atlantic grenadier	+	+	+	(6)
Noemacheilus barbatulus	stone loach	+	+	+	(6)
Oblada melanura	saddled seabream	+	+	-	(6)
Odontesthes bonariensis	Argentine silverside	+	+	*	(22)
Oreochromis niloticus	tilapia	+	+	*	(42,43)

Oreochromis niloticus/O. aureus	tilapia hybrid	+	+	*	(10)
Parophrys vetulus	English sole	*	*	+	(44)
Perca flavescens	yellow perch	*	+	*	(45)
Perca fluviatilis	perch	+	+	+	(46)
Peristedion cataphractum	African armored searobin	+	+	-	(6,9)
Pholis gunnellus	rock gunnel (butterfish)	+	+	-	(6)
Phycis blennoides	greater fork-beard	+	+	-	(6)
Pimelodus maculatus	spotted pim	*	+	+	(23)
Platichthys stellatus	starry flounder	+	*	+	(47)
Pleuronectes flesus	flounder	+	*	*	(12)
Pleuronectes microcephalus	dab (lemon dab?)	+	*	*	(12)
Pleuronectes platessa	plaice	+	+	+	(6,9,10,12,13,48-51)
Poecilia	molly genus	+	+	+	(6)
Polyodon spathula	American paddlefish	+	+	+	(6)
Polypterus senegalis	Senegal bichir	+	+	+	(6)
Prochilodus argenteus	curimatá-pacu	+	*	+	(52)
Prochilodus scrofa	curimatá	+	+	+	(23)
Protopterus aethiopicus	marbled lungfish	+	+	+	(6)
Pseudopleuronectes americanus	winter flounder	+	*	+	(53)
Raia clavata	thornback ray	-	-	+	(6)
Rhombus maximus	turbot	+	+	+	(9,13,54)
Rivulus marmoratus	mangrove rivulus	*	*	+	(55)
Rutilus rutilus	common roach	+	+	+	(13,46,56)
Salmo gairdneria	rainbow trout	+	+	+	(6,9,10,51,57)
Salmo salar	Atlantic salmon	+	+	+	(6,58-61)
Salmo trutta	brown trout	+	+	+	(6)
Sardina pilchardus	sardine	+	*	*	(62)
Sarotherodon mossambicus	Mozambique talapia	+	+	-	(9)
Scleropages formosus	Asian arowana	+	+	+	(6)
Scophthalmus maximus	turbot	+	+	-	(6,9,54,63,64)
Scyliorhinus canicula	spotted cat shark/dogfish	+	-	+	(6,10,51)
Sebastes	rockfish genus	+	+	+	(6)
Serranus scriba	painted comber	+	+	+	(6)
Solea	sole genus	+	*	*	(8)
Solea senegalensis	Senegalese sole	+	+	*	(22)
Sparus aurata	gilthead seabream	+	+	+	(24,65)
Squalus acanthias	spiny dog fish	-	-	+	(6)
Tachysurus (formerly *Tachyrus)*	bagrid catfish genus	+	*	*	(8)
Tautogolabrus adspersus	cunner	+	+	+	(66)
Tetraodon	pufferfish genus	+	+	+	(6)
Thunnus maccoyii	Southern bluefin tuna	+	+	+	(67)
Thunnus thynnus L.	Atlantic bluefin tuna	*	*	+	(68)
Tilapia mariae	spotted tilapia	+	+	+	(6)
Tilapia mossambica	mozambique tilapia	+	+	+	(69)

Tilapia zillii	redbelly tilapia	+	+	*	(51)
Trachinus	weever genus	+	+	+	(6)
Trachurus trachurus	Atlantic horse mackerel	+	+	+	(6)
Trachyrhynchus murrayi	roughnose grenadier	+	+	+	(6)
Trichogaster leeri	pearl fouramey	+	+	*	(70)
Trigla gurnardus	grey gunnard	+	*	*	(12,13)
Trisopterus esmarki	Norway pout	+	+	+	(6)
Trisopterus minutus	poor cod	+	+	+	(6)
Xiphophorus helleri	green swordtail	+	+	-	(9,51)
Xiphophorus maculatus	platyfish	+	+	+	(70,71)
Zeus	dory genus	+	*	*	(8)
Zeus faber	John dory	+	+	+	(6,9)
Zoarces viviparus	European eelpout	+	+	-	(6)

Supplemental Table 2. Amphibian and reptile species with MMCs reported in the spleen, kidney, and/or liver. +: MMCs present in tissue, -: MMCs absent in tissue, *: MMC status has not been determined for this tissue.

Species	Common name	Spleen	Kidney	Liver	Reference
**AMPHIBIANS**					
Ambystoma mexicanum	axolotl	*	*	+	(72)
Ambystoma tigrinum mavortium	barred tiger salamander	+	+	+	(73)
Amphiuma means	two-toed amphiuma	*	*	+	(74)
Bufo cognatus	Great Plains toad	+	+	+	(73)
Eupemphix/Physalaemus nattereri	Cuyaba dwarf frog	+	*	+	(75-78)
Fejervarya limnocharis	Indian cricket frog	*	*	+	(79)
Hoplobatrachus occipitalis	crowned bullfrog	*	*	+	(80)
Hoplobatrachus rugulosus	Chinese edible frog	+	*	+	(81)
Leptodactylus latinasus	urnero	*	*	+	(78)
Notophthalmus viridescens	eastern newt	*	*	+	(82)
Rana clamitans	bronze frog	*	*	+	(83)
Rana esculenta L.	edible frog	+	*	+	(72,84-87)
Rana palustris	pickerel frog	*	*	+	(83)
Rana pipiens	norther leopard frog	*	*	+	(83)
Rana ridibunda	marsh frog	*	*	+	(88)
Rana sylvatica	wood frog	*	*	+	(83)
Spea multiplicata	New Mexico spadefoot toad	+	+	+	(73)
Triturus a. apuanus	Italian alpine newt	*	*	+	(85)
Triturus carnifex	Italian crested newt	*	*	+	(85)
Triturus cristatus Laurenti	great crested newt	*	*	+	(72)
Xenopus laevis	African clawed frog	+	*	+	(72,89)
**REPTILES**					
Agama stellio	starred agama	*	*	+	(90)
Alligator mississippiensis	American alligator	*	*	+	(91)
Apalone mutica	smooth softshell turtle	+	*	+	(92)
Chelydra serpentina	common snapping turtle	+	*	+	(92)
Chrysemys picta bellii	painted turtle	+	*	+	(92)
Crocodylus niloticus	Nile crocodile	*	*	+	(93)
Emydoidea blandingii	Blanding's turtle	+	*	+	(92)
Gekko japonicas	Schlegel's Japanese gecko	*	*	+	(91)
Hemidactylus frenatus	common house gecko	*	*	+	(91)
Hydrophis melanocephalus	slender-necked sea snake	*	*	+	(91)
Japalura polygonata ishigakiensis	Okinawa tree lizard	*	*	+	(91)
Kinosternon flavescens	yellow mud turtle	+	*	+	(94)
Mauremys japonica	Japanese pond turtle	*	*	+	(91)
Mauremys reevesii	Chinese pond turtle	*	*	+	(91)
Pelodiscus sinensis	Chinese softshell turtle	*	*	+	(91)
Phrynops geoffroanus	Geoffroy's toadhead turtle	*	*	+	(95)
Plestidon japonicas	Japanese skink	*	*	+	(91)
Plestiodon kishinouyei	Kishinoue's giant skink	*	*	+	(91)

Psammophis sibilans	hissing sand snake	+	*	*	(96)
Scincella boettgeri	smooth skink	*	*	+	(91)
Takydromus dorsalis	Sakishima grass lizard	*	*	+	(91)
Takydromus smaragdinus	Japanese green grass lizards	*	*	+	(91)
Takydromus tachydromoides	Japanese grass lizard	*	*	+	(91)
Terrapene Carolina	common box turtle	*	*	+	(97)
Testudo graeca L	Mediterranean turtle	*	*	+	(84)
Trachemys scripta elegans	red-eared slider turtle	*	*	+	(91)

Supplemental Table 3. Fish and reptile species reported lacking MMCs. -: MMCs absent in tissue, *: MMC status has not been determined for this tissue.

Species	Common name	Spleen	Kidney	Liver	Reference
**FISH**					
Hypostomus facisci	South American armored catfish	-	*	-	(29)
Lampetra fluviatilis	European river lamprey	*	-	-	(6)
Spinachia vulgaris	15 spined stickleback	-	*	*	(12)
**REPTILES**					
Dinodon orientale	Oriental odd-tooth snake	*	*	-	(91)
Elaphe climacophora	Japanese rat snake	*	*	-	(91)
Elaphe quadrivirgata	Japanese striped snake	*	*	-	(91)
Elaphe taeniura schmackerii	beauty rat snake	*	*	-	(91)
Gloydius blomhofii	mamushi	*	*	-	(91)
Hebius vibakari	Asian keelback	*	*	-	(91)
Protobothrops elegans	lace-headed viper	*	*	-	(91)
Rhabdophis tigrinus	tiger keelback	*	*	-	(91)

References for Supplemental Materials

1. Rabosky DL, Santini F, Eastman J, Smith SA, Sidlauskas B, Chang J, Alfaro ME. Rates of speciation and morphological evolution are correlated across the largest vertebrate radiation. *Nature Communications* (2013) **4**:1–8. doi:10.1038/ncomms2958

2. Paradis E, Claude J, Strimmer K. APE: Analyses of Phylogenetics and Evolution in R language. *Bioinformatics* (2004) **20**:289–290. doi:10.1093/bioinformatics/btg412

3. Pyron RA, Wiens JJ. Molecular Phylogenetics and Evolution. *Molecular Phylogenetics and Evolution* (2011) **61**:543–583. doi:10.1016/j.ympev.2011.06.012

4. Zheng Y, Wiens JJ. Combining phylogenomic and supermatrix approaches, and a time-calibrated phylogeny for squamate reptiles (lizards and snakes) based on 52 genes and 4162 species. *Molecular Phylogenetics and Evolution* (2015) **94**:537–547. doi:10.1016/j.ympev.2015.10.009

5. Rosindell J, Harmon LJ. OneZoom: A Fractal Explorer for the Tree of Life. *PLoS Biol* (2012) **10**:e1001406. doi:10.1371/journal.pbio.1001406.s003

6. Agius C. Phylogenetic development of melano–macrophage centres in fish. *Journal of zoology* (1980) **191**:11–31.

7. Fournie JW, Summers JK, Courtney LA, Engle VD, Blazer VS. Utility of Splenic Macrophage Aggregates as an Indicator of Fish Exposure to Degraded Environments. *Journal of Aquatic Animal Health* (2001) **13**:105–116. doi:10.1016/0145-305X(91)90007-L

8. Blumenthal R. Sur le rôle érythrolytique de la rate chez les Poissons. *Comptes rendus hebdomadaires des seances de lAcademie des science* (1908) **146**:190–191.

9. Agius C. The role of melano-macrophage centres in iron storage in normal and diseased fish. *Jornal of Fish Diseases* (1979) **2**:337–343.

10. Agius C, Agbede SA. An electron microscopical study on the genesis of lipofuscin, melanin and haemosiderin in the haemopoietic tissues of fish. *Journal of Fish Biology* (1984) **24**:471–488.

11. Lamers CH, De Haas MJ. Antigen localization in the lymphoid organs of carp (Cyprinus carpio). *Cell Tissue Res* (1985) **242**:491–498.

12. Yoffey JM. A Contribution to the Study of the Comparative Histology and Physiology of the Spleen, with reference chiefly to its Cellular Constituents: I. In Fishes. *J Anat* (1929) **63**:314–344.5.

13. Roberts RJ. *The Pathology of Fishes*. eds.W. E. Ribelin, G. Migaki Univ of Wisconsin Press (1975).

14. Herráez MP, Zapata AG. Structure and function of the melano-macrophage centres of the goldfish Carassius auratus. *Veterinary Immunology and Immunopathology* (1986) **12**:117–126.

15. Ziegenfuss MC, Wolke RE. The Use of Fluorescent Microspheres in the Study of Piscine Macrophage Aggregate Kinetics. *Developmental & Comparative Immunology* (1991) **15**:165–171.

16. Diaz-Satizabal L, Magor BG. Isolation and cytochemical characterization of melanomacrophages and melanomacrophage clusters from goldfish (*Carassius auratus,* L.). *Developmental & Comparative Immunology* (2015) **48**:221–228. doi:10.1016/j.dci.2014.10.003

17. Couillard CM, Hodson PV. Pigmented macrophage aggregates: A toxic response in fish exposed to bleached-kraft mill effluent? *Environmental Toxicology and Chemistry* (1996) **15**:1844–1854.

18. Verma VK, Rani KV, Sehgal N, Prakash O. Prevention of histopathological damages in the liver, spleen and kidney of Channa punctata infected with Aeromonas hydrophila. *DU Journal of Undergraduate Research and Innovation* (2016) **2**:227–232.

19. Sayed AH, Younes HAM. Melanomacrophage centers in Clarias gariepinus as an immunological biomarker for toxicity of silver nanoparticles. *Journal of Microscopy and Ultrastructure* (2016) **5**:97–104. doi:10.1016/j.jmau.2016.07.003

20. Secombes CJ. Histological Changes in Lymphoid Organs of Carp Following Injection of Soluble or Particulate Antigens. *Developmental & Comparative Immunology* (1982) **Supplement 2**:53–58.

21. Lamers CH. Histophysiology of a primary immune response against Aeromonas hydrophila in carp (Cyprinus carpio L.). *Journal of Experimental Zoology* (1986) **238**:71–80.

22. Vigliano FA, Bermúdez R, Quiroga MI, Nieto JM. Evidence for melano-macrophage centres of teleost as evolutionary precursors of germinal centres of higher vertebrates: An immunohistochemical study. *Fish and Shellfish Immunology* (2006) **21**:467–471. doi:10.1016/j.fsi.2005.12.012

23. Tokumaru M, Ferri AG. Histochemistry of the pigments found in the liver, spleen and kidney of the fresh-water fish. *Rev Bras Biol* (1970) **30**:163–171.

24. Meseguer J, Lopez-Ruiz A, Esteban MA. Melano-macrophages of the seawater teleosts, sea bass (Dicentrarchus labrax) and gilthead seabream (Sparus aurata): morphology, formation and possible function. *Cell Tissue Res* (1994) **277**:1–10.

25. Kurtović B, Teskeredžić E, Teskeredžić Z. Histological comparison of spleen and kidney tissue from farmed and wild European sea bass (Dicentrarchus labrax L.). *Acta Adriatica* (2008) **49**:147–154.

26. Lin HT, Lin HY, Yang HL. Histology and histochemical enzyme-staining patterns of major immune organs in Epinephelus malabaricus. *Journal of Fish Biology* (2005) **66**:729–740. doi:10.1111/j.1095-8649.2005.00635.x

27. Abdelsalam M, Abdel-Gaber R, Mahmoud MA, Mahdy OA, Khafaga NIM, Warda M. Morphological, molecular and pathological appraisal of Callitetrarhynchus gracilis plerocerci (Lacistorhynchidae) infecting Atlantic little tunny (Euthynnus alletteratus) in Southeastern Mediterranean. *Journal of Advanced Research* (2016) **7**:317–326. doi:10.1016/j.jare.2015.07.004

28. Blazer VS, Fournie JW, Weeks-Perkins BA. Macrophage aggregates: biomarker for immune function in fishes? *Environmental Toxicology and Risk Assessment (Sixth Volume)* (1997)360–375.

29. Sales CF, Silva RF, Amaral MGC, Domingos FFT, Ribeiro RIMA, Thomé RG, Santos HB. Comparative histology in the liver and spleen of three species of freshwater teleost. *Neotrop ichthyol* (2017) **15**: doi:10.1590/1982-0224-20160041

30. Kranz H, Peters N. Melano-Macrophage Centers in Liver and Spleen of Ruffe (Gymnocephalus-Cernua) From the Elbe Estuary. *Helgolander Meeresuntersuchungen* (1984) **37**:415–424.

31. Kumar R, Joy KP, Singh SM. Morpho-histology of head kidney of female catfish Heteropneustes fossilis: seasonal variations in melano- macrophage centers, melanin contents and effectsof

lipopolysaccharide and dexamethasone on melanins. *Fish Physiol Biochem* (2016) **42**:1287–1306. doi:10.1007/s10695-016-0218-2

32. Tsujii T, Seno S. Melano-macrophage centers in the aglomerular kidney of the sea horse (teleosts): morphologic studies on its formation and possible function. *Anat Rec* (1990) **226**:460–470. doi:10.1002/ar.1092260408

33. Mela M, Randi MAF, Ventura DF, Carvalho CEV, Pelletier E, Oliveira Ribeiro CA. Effects of dietary methylmercury on liver and kidney histology in the neotropical fish Hoplias malabaricus. *Ecotoxicology and Environmental Safety* (2007) **68**:426–435. doi:10.1016/j.ecoenv.2006.11.013

34. Saunders HL, Oko AL, Scott AN, Fan CW, Magor BG. The cellular context of AID expressing cells in fish lymphoid tissues. *Developmental & Comparative Immunology* (2010) **34**:669–676. doi:10.1016/j.dci.2010.01.013

35. Kranz H. Changes in Splenic Melano-Macrophage Centers of Dab Limanda-Limanda During and After Infection with Ulcer Disease. *Diseases of Aquatic Organisms* (1989) **6**:167–173.

36. BUCKE D, VETHAAK AD, LANG T. Quantitative Assessment of Melanomacrophage Centers (Mmcs) in Dab Limanda-Limanda Along a Pollution Transect in the German Bight. *Marine Ecology Progress Series* (1992) **91**:193–196.

37. Elarifi AE. The histopathology of larval anisakid nematode infections in the liver of whiting, Merlangius merlangus (L.), with some observations on blood leucocytes of the fish. *J Fish Dis* (1982) **5**:411–419.

38. Huizinga HW, Esch GW, Hazen TC. Histopathology of red‐sore disease (Aeromonas hydrophila) in naturally and experimentally infected largemouth bass Micropterus salmoides (Lacepede). *J Fish Dis* (1979) **2**:263–277.

39. Blazer VS, Wolke RE, Brown J, Powell CA. Piscine Macrophage Aggregate Parameters as Health Monitors - Effect of Age, Sex, Relative Weight, Season and Site Quality in Largemouth Bass (Micropterus-Salmoides). *Aquatic Toxicology* (1987) **10**:199–215.

40. Pierce KV, McCain B, Sherwood MJ. Histology of liver tissue from Dover sole. *Coastal Water Research Project* (1977)

41. Bunton TE, Baksi SM, George CJ, Frazier JM. Abnormal Hepatic Copper Storage in a Teleost Fish (Morone, Americana). *Vet Pathol* (1987) **24**:515–524. doi:10.1177/030098588702400608

42. Manrique WG, da Silva Claudiano G, Petrillo TR, de Castro MP, Pereira Figueiredo MA, de Andrade Belo MA, de Moraes JRE, de Moraes FR. Response of splenic melanomacrophage centers of Oreochromis niloticus (Linnaeus, 1758) to inflammatory stimuli by BCG and foreign bodies. *J Appl Ichthyol* (2014) **30**:1001–1006. doi:10.1111/jai.12445

43. Wen C-M. Development and characterization of a cell line from tilapia head kidney with melanomacrophage characteristics. *Fish and Shellfish Immunology* (2016) **49**:442–449. doi:10.1016/j.fsi.2016.01.013

44. Pierce KV, McCain BB, Wellings SR. Pathology of hepatomas and other liver abnormalities in English sole (Parophrys vetulus) from the Duwamish River estuary, Seattle, Washington. *J Natl Cancer Inst* (1978) **60**:1445–1453.

45. Brown CL, George CJ. Age-Dependent Accumulation of Macrophage Aggregates in the Yellow Perch, Perca-Flavescens (Mitchill). *J Fish Dis* (1985) **8**:135–138.

46. Haaparanta A, Valtonen ET, Hoffmann R, Holmes J. Do macrophage centres in freshwater

fishes reflect the differences in water quality? *Aquatic Toxicology* (1996) **34**:253–272.

47. Pierce KV, McCain BB, Wellings SR. Histopathology of abnormal livers and other organs of starry flounder Platichthys stellatus (Pallas) from the estuary of the Duwamish River, Seattle, Washington, USA. *J Fish Dis* (1980) **3**:81–91.

48. Ellis AE, De Sousa M. Phylogeny of the lymphoid system. I. A study of the fate of circulating lymphocytes in plaice. *Eur J Immunol* (1974) **4**:338–343. doi:10.1002/eji.1830040505

49. Ellis AE, Munroe A, Roberts RJ. Defence mechanisms in fish. 1. A study of the phagocytic system and the fate of intraperitoneally injected particulate material in the plaice (*Pleuronectes platessa* L.). *Journal of Fish Biology* (1976) **8**:67–79.

50. Ellis AE. Antigen‐trapping in the spleen and kidney of the plaice Pleuronectes platessa L. *J Fish Dis* (1980) **3**:413–426.

51. Agius C, Roberts RJ. Effects of starvation on the melano‐macrophage centres of fish. *Journal of Fish Biology* (1981) **19**:161–169.

52. Ribeiro HJ, Procópio MS, Gomes JMM, Vieira FO, Russo RC, Balzuweit K, Chiarini-Garcia H, Santana Castro AC, Rizzo E, Corrêa JD. Functional dissimilarity of melanomacrophage centres in the liver and spleen from females of the teleost fish Prochilodus argenteus. *Cell Tissue Res* (2011) **346**:417–425. doi:10.1007/s00441-011-1286-3

53. Wolke RE, George CJ, Blazer VS. Pigmented macrophage accumulations (MMC; PMB): possible monitors of fish health. ed.J. W. Hargis National Marine Fishery Service, Washington, DC (1985).

54. Ferguson HW. The relationship between ellipsoids and melano-macrophage centres in the spleen of turbot (Scophthalmus maximus). *Journal of Comparative Pathology* (1976) **86**:377–380.

55. Vogelbein WK, Fournie JW. Sequential development and morphology of experimentally induced hepatic melano‐macrophage centres in Rivulus marmoratus. *Journal of Fish Biology* (1987) **31**:145–153.

56. Pronina SV, Batueva MDD, Pronin NM. Characteristics of melanomacrophage centers in the liver and spleen of the roach Rutilus rutilus (Cypriniformes: Cyprinidae) in Lake Kotokel during the Haff disease outbreak. *J Ichthyol* (2014) **54**:104–110. doi:10.1134/S003294521401010X

57. Passantino L, Cianciotta A, Jirillo F, Carrassi M, Jirillo E, Passantino GF. Lymphoreticular System in Fish: Erythrocyte-Mediated Immunomodulation of Macrophages Contributes to the Formation of Melanomacrophage Centers. *Immunopharmacology & Immunotoxicology* (2005) **27**:147–161. doi:10.1081/IPH-200051766

58. Press CM, Dannevig BH, Landsverk T. Immune and enzyme histochemical phenotypes of lymphoid and nonlymphoid cells within the spleen and head kidney of Atlantic salmon (*Salmo salar* L.). *Fish and Shellfish Immunology* (1994) **4**:79–93.

59. Falk K, Press CM, Landsverk T, Dannevig BH. Spleen and kidney of Atlantic salmon (Salmo salar L.) show histochemical changes early in the course of experimentally induced infectious salmon anaemia (ISA). *Veterinary Immunology and Immunopathology* (1995) **49**:115–126.

60. Brattgjerd S, Evensen O. A sequential light microscopic and ultrastructural study on the uptake and handling of Vibrio salmonicida in phagocytes of the head kidney in experimentally infected Atlantic salmon (Salmo salar L.). *Vet Pathol* (1996) **33**:55–65.

61. Sissener NH, Bakke AM, Gu J, Penn MH, Eie E, Krogdahl A, Sanden M, Hemre GI. An assessment of organ and intestinal histomorphology and cellular stress response in Atlantic salmon (Salmo salar L.) fed genetically modified Roundup Ready (R) soy. *Aquaculture* (2009) **298**:101–110. doi:10.1016/j.aquaculture.2009.10.011

62. Beveren E, Keck N, Fromentin J-M, Laurence S, Boulet H, Labrut S, Baud M, Bigarré L, Brosset P, Saraux C. Can pathogens alter the population dynamics of sardine in the NW Mediterranean? *Marine Biology* (2016) **163**:1–9. doi:10.1007/s00227-016-3015-7

63. Fournier-Betz V, Quentel C, Lamour F, Leven A. Immunocytochemical detection of Ig-positive cells in blood, lymphoid organs and the gut associated lymphoid tissue of the turbot (Scophthalmus maximus). *Fish and Shellfish Immunology* (2000) **10**:187–202. doi:10.1006/fsim.1999.0235

64. Bermúdez R, Vigliano F, Marcaccini A, Sitjà-Bobadilla A, Quiroga MI, Nieto JM. Response of Ig-positive cells to Enteromyxum scophthalmi (Myxozoa) experimental infection in turbot, Scophthalmus maximus (L.): A histopathological and immunohistochemical study. *Fish and Shellfish Immunology* (2006) **21**:501–512. doi:10.1016/j.fsi.2006.02.006

65. Montero D, Blazer VS, Socorro J, Izquierdo MS, Tort L. Dietary and culture influences on macrophage aggregate parameters in gilthead seabream (Sparus aurata) juveniles. *Aquaculture* (1999) **179**:523–534.

66. Mackmull G, Michels NA. Absorption of colloidal carbon from the peritoneal cavity in the teleost, Tautogolabrus adspersus. *American Journal of Anatomy* (1932) **51**:3–47.

67. Evans D, Nowak B. Effect of ranching time on melanomacrophage centres in anterior kidney and spleen of Southern bluefin tuna, Thunnus maccoyii. *Fish and Shellfish Immunology* (2016) **59**:358–364. doi:10.1016/j.fsi.2016.11.014

68. Passantino L, Santamaria N, Zupa R, Pousis C, Garofalo R, Cianciotta A, Jirillo E, Acone F, Corriero A. Liver melanomacrophage centres as indicators of Atlantic bluefin tuna, Thunnus thynnusL. well-being. *J Fish Dis* (2013) **37**:241–250. doi:10.1111/jfd.12102

69. Suresh N. Effect of cadmium chloride on liver, spleen and kidney melano macrophage centres in Tilapia mossambica. *J Environ Biol* (2009) **30**:505–508.

70. Leknes IL. Melano-macrophage centres and endocytic cells in kidney and spleen of pearl gouramy and platyfish (Anabantidae, Poeciliidae: Teleostei). *Acta Histochemica* (2007) **109**:164–168. doi:10.1016/j.acthis.2006.10.003

71. Leknes IL. Melano-macrophage centres in the liver of platyfish, Xiphophorus maculatus, Poeciliidae: Teleostei. *Zoology* (2004) **107**:201–204. doi:10.1016/j.zool.2004.07.002

72. Sichel G, Scalia M, Mondio F, Corsaro C. The amphibian Kupffer cells build and demolish melanosomes: an ultrastructural point of view. *Pigment Cell Res* (1997) **10**:271–287.

73. Miller DL, Miller DL, Bursey CR, Gray MJ, Smith LM. Metacercariae of *Clinostomum attenuatum* in *Ambystoma tigrinum mavortium*, *Bufo cognatus* and *Spea multiplicata* from west Texas. *j helminthol* (2004) **78**:373–376. doi:10.1079/JOH2004248

74. Hack MH, Helmy FM. An Analysis of Melanoprotein from Amphiuma Liver and from a Human Liver Melanoma. *Proc Soc Exp Biol Med* (1964) **116**:348–350.

75. Franco-Belussi L, de Lauro Castrucci AM, de Oliveira C. Responses of melanocytes and melanomacrophages of Eupemphix nattereri (Anura: Leiuperidae) to Nle4, D-Phe7-α-melanocyte stimulating hormone and lipopolysaccharides. *Zoology* (2013) **116**:316–324.

doi:10.1016/j.zool.2013.06.003

76. De Souza Santos LR, Franco-Belussi L, Zieri R, Borges RE, de Oliveira C. Effects of Thermal Stress on Hepatic Melanomacrophages of Eupemphix nattereri(Anura). *Anat Rec* (2014) **297**:864–875. doi:10.1002/ar.22884

77. Franco-Belussi L, De Oliveira C. The spleen of Physalaemus nattereri (Amphibia: Anura): morphology, melanomacrophage pigmentcompounds and responses to alpha-melanocyte stimulating hormone. *Italian Journal of Zoology* (2016) **83**:298–305. doi:10.1080/11250003.2016.1194488

78. Pérez-Iglesias JM, Franco-Belussi L, Moreno L, Tripole S, de Oliveira C, Natale GS. Effects of glyphosate on hepatic tissue evaluating melanomacrophages and erythrocytes responses in neotropical anuran Leptodactylus latinasus. *Environ Sci Pollut Res* (2016) **23**:9852–9861. doi:10.1007/s11356-016-6153-z

79. Jantawongsri K, Thammachoti P, Kitana J, Khonsue W, Varanusupakul P, Kitana N. Altered Immune Response of the Rice Frog Fejervarya limnocharis Living in Agricultura Area with Intensive Herbicide Utilization at Nan, Providence, Thailand. *EnvironmentAsia* (2015) **8**:68–74.

80. Olayemi OA, Jagun JA, Felix AO, Adewole AA. Cadmium Bio-Accumulation and the Associated Biomarkers in Edible Frog Species (Hoplobatrachus Occipitalis) in Ibadan, Oyo State, Nigeria. *World's Veterinary Journal* (2016) **6**:70–79.

81. Sailasuta A, Satetasit J, Chutmongkonkul M. Pathological Study of Blood Parasites in Rice Field Frogs, Hoplobatrachus rugulosus(Wiegmann, 1834). *Veterinary Medicine International* (2011) **2011**:1–5. doi:10.1016/S0065-308X(08)60184-0

82. Goldblatt PJ, Hampton JA, DiDio LN, Skeel KA, Klaunig JE. Morphologic and histochemical analysis of the newt (Notophthalmus viridescens) liver. *Anat Rec* (1987) **217**:328–338. doi:10.1002/ar.1092170403

83. Rohr JR, Schotthoefer AM, Raffel TR, Carrick HJ, Halstead N, Hoverman JT, Johnson CM, Johnson LB, Lieske C, Piwoni MD, et al. Agrochemicals increase trematode infections in a declining amphibian species. *Nature* (2008) **455**:1235–1239. doi:10.1038/nature07281

84. Scalia M, GEREMIA E, Corsaro C, SANTORO C, Sciuto S, Sichel G. The Extracutaneous Pigmentary System - Evidence for the Melanosynthesis in Amphibia and Reptilia Liver. *Comparative Biochemistry and Physiology B-Biochemistry & Molecular Biology* (1988) **89**:715–717.

85. Barni S, Bertone V, Croce AC. Increase in liver pigmentation during natural hibernation in some amphibians. *J Anat* (1999) **195**:19–25.

86. Barni S, Vaccarone R, Bertone V, Fraschini A. Mechanisms of changes to the liver pigmentary component during the annual cycle (activity and hibernation) of Rana esculenta L. *J Anat* (2002) **200**:185–194.

87. Gallone A, Guida G, Maida I, Cicero R. Spleen and liver pigmented macrophages of Rana esculenta L. - A new melanogenic system? *Pigment Cell Res* (2002) **15**:32–40.

88. Paunescu A, Ponepal CM, Drghici O. Liver histopathologic alterations in the frog Rana (Pelophylax) Ridibunda induce by the action of reldan 40EC insecticide. *An UO Fasc Biol* (2010) **17**:166–169.

89. Zuasti A, Jiménez-Cervantes C, García-Borrón JC, Ferrer C. The melanogenic system of Xenopus laevis. *Arch Histol Cytol* (1998) **61**:305–316.

90. Paperna I, Kremer-Mecabell T, Finkelman S. Hepatozoon kisraen. sp. infecting the lizard Agama stelliois transmitted by the tick Hyalommacf. aegyptium. *Parasite* (2014) **9**:17–27. doi:10.1051/parasite/200209117

91. Akiyoshi H, Inoue-Matsuo A. Comparative Histological Study of Parenchymal Arrangements in Three Orders of Reptilian Livers. *J Phylogen Evolution Biol* (2016) **4**: doi:10.4172/2329-9002.1000161

92. Johnson JC, Schwiesow T, Ekwall AK, Christiansen JL. Reptilian melanomacrophages function under conditions of hypothermia: observations on phagocytic behavior. *Pigment Cell Res* (1999) **12**:376–382.

93. van Wilpe E, Groenewald HB. Kupffer cell structure in the juvenile Nile crocodile, Crocodylus niloticus. *Journal of Morphology* (2013) **275**:1–8. doi:10.1002/jmor.20188

94. Christiansen JL, Grzybowski JM, Kodama RM. Melanomacrophage aggregations and their age relationships in the yellow mud turtle, Kinosternon flavescens (Kinosternidae). *Pigment Cell Res* (1996) **9**:185–190.

95. Moura LR, Santos A, Belleti ME, Vieira LG. Morphological aspects of the liver of the freshwater turtle Phrynops geoffroanus Schweigger, 1812 (Testudines, Chelidae). *Brazilian Journal of Morphology* (2009) **26**:129–134.

96. Saad AH. Ultrastructure of Phagocytic Cells in the Spleen of *Psammophils silbans* (Serpentes: Colubridae). *Journal of Morphology* (1994) **222**:231–240.

97. Henninger JM, Beresford WA. Is it coincidence that iron and melanin coexist in hepatic and other melanomacrophages? *Histol Histopathol* (1990) **5**:457–459.
